# Subtyping of COVID-19 samples based on cell-cell interaction in single cell transcriptomes

**DOI:** 10.1038/s41598-023-46350-2

**Published:** 2023-11-10

**Authors:** Kyeonghun Jeong, Yooeun Kim, Jaemin Jeon, Kwangsoo Kim

**Affiliations:** 1 Interdisciplinary Program in Bioengineering, Seoul National University, Seoul, 08826 Republic of Korea; 2 Interdisciplinary Program in Bioinformatics, Seoul National University, Seoul, 08826 Republic of Korea; 3 Department of Transdisciplinary Medicine, Institute of Convergence Medicine with Innovative Technology, Seoul National University Hospital, Seoul, 03080 Republic of Korea; 4 Department of Medicine, Seoul National University, Seoul, 03080 Republic of Korea

**Keywords:** Immunology, Molecular biology, Molecular medicine, Computational biology and bioinformatics, Cellular signalling networks, Functional clustering

## Abstract

In single-cell transcriptome analysis, numerous biomarkers related to COVID-19 severity, including cell subtypes, genes, and pathways, have been identified. Nevertheless, most studies have focused on severity groups based on clinical features, neglecting immunological heterogeneity within the same severity level. In this study, we employed sample-level clustering using cell-cell interaction scores to investigate patient heterogeneity and uncover novel subtypes. The clustering results were validated using external datasets, demonstrating superior reproducibility and purity compared to gene expression- or gene set enrichment-based clustering. Furthermore, the cell-cell interaction score-based clusters exhibited a strong correlation with the WHO ordinal severity score based on clinical characteristics. By characterizing the identified subtypes through known COVID-19 severity-associated biomarkers, we discovered a “Severe-like moderate” subtype. This subtype displayed clinical features akin to moderate cases; however, molecular features, such as gene expression and cell-cell interactions, resembled those of severe cases. Notably, all patients who progressed from moderate to severe belonged to this subtype, underscoring the significance of cell-cell interactions in COVID-19 patient heterogeneity and severity.

## Introduction

Coronavirus-19 (COVID-19) is an infectious disease that has had a great impact on humanity, as it has caused about 6.5 billion confirmed cases and about 6 million deaths internationally, and about 28 million confirmed cases and about 30,000 deaths in Korea^1^. When infected with COVID-19, mild or moderate patients only have symptoms such as fever, cough, and fatigue, but in severe patients, pneumonia and organ damage can lead to death^2^.

The body’s immune system recognizes antigens to generate antibodies, and in the process of activating the immune system in response, collects immune cells, secretes cytokines, and responds. Cell-to-cell interaction is essential for all of these immune responses. Protein-related techniques are needed to accurately capture these cell-cell interactions, but because the transcriptome data is much larger and easier to analyze, several methodologies are emerging to infer cell-cell interactions^3^.

According to previous studies, it is suggested that the difference in immune response plays an important role in the difference in symptoms between mild and severe symptoms^4^. One study explored the heterogeneity of cell-cell interactions in different tissues among moderate and severe COVID-19 patients^5^. Another study primarily conducted their analysis at the cell or cell type level^6^. While this approach provides valuable cellular insights, it may not fully capture the heterogeneity at the sample level, which is essential for a direct correlation with clinical traits and symptoms. Therefore, our study places a particular emphasis on sample-level analysis. A further study used proteomics to delve into the heterogeneity within severe COVID-19 patients^7^.

Despite active research to identify markers of severity, existing studies have often not placed sufficient emphasis on observing heterogeneity between samples within the same severity groups. In contrast to previous work that mainly focused on severity labels, our study aims to uncover the molecular heterogeneity within the same severity group. We hypothesized that even within severity groups divided based on clinical variables, samples would exhibit unique molecular properties. To substantiate this, our large-scale study aimed to uncover reproducible clusters associated with severity, thereby emphasizing the critical role of molecular heterogeneity. We believe that nuanced molecular variations within each severity group are still noticeable and significant. Relying solely on severity groups can mask these subtle molecular distinctions, as these classifications often reflect average molecular traits. By focusing on severity-associated features, we aim to highlight these nuanced molecular variations that might otherwise be overlooked. This approach not only deepens our understanding of molecular subtleties but also bridges the gap between clinical assessments and the inherent molecular complexities of the disease.

**Figure 1:**
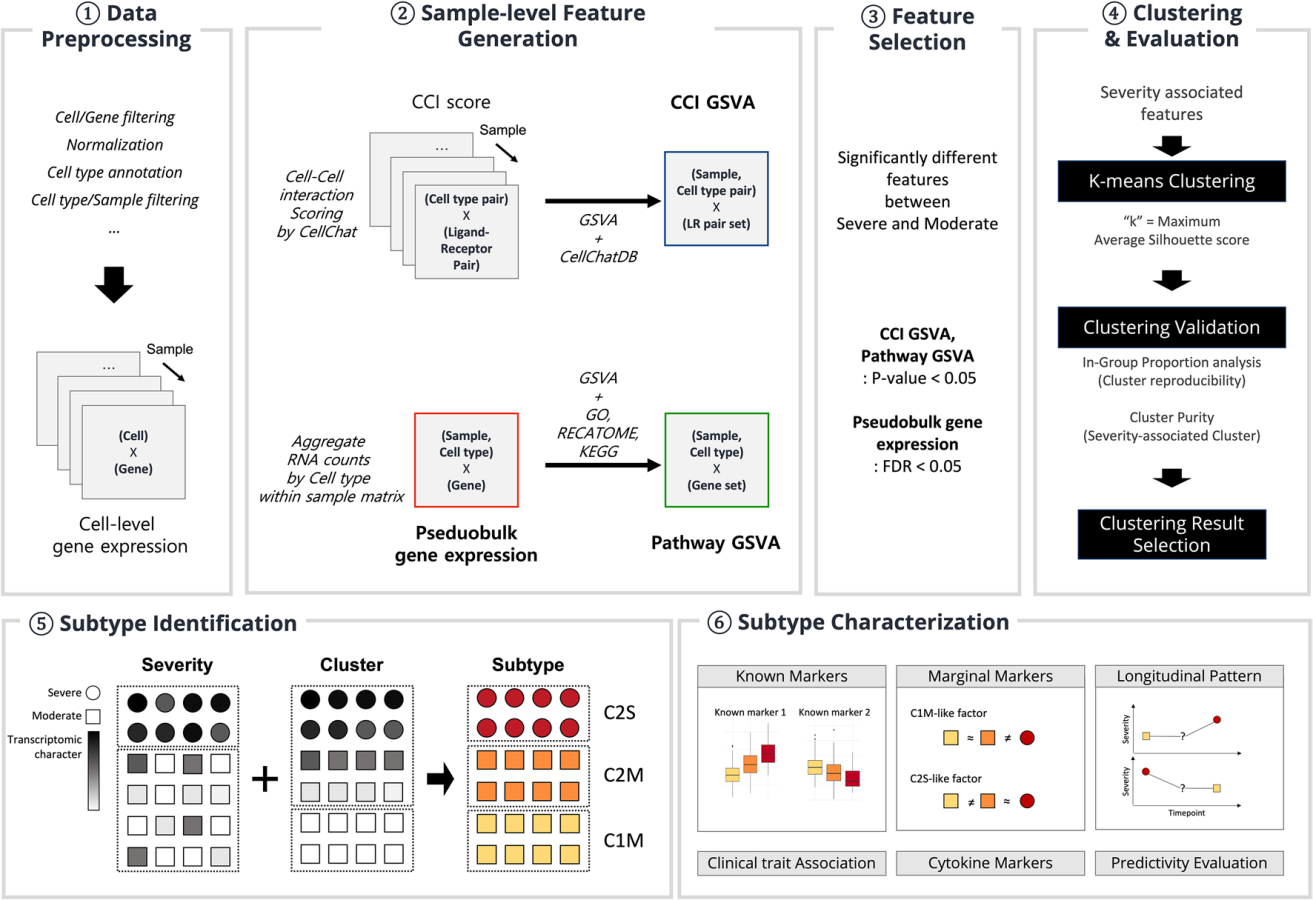
Summary of the overall analysis workflow: After preprocessing single-cell RNA sequencing data, sample-level features were generated. From the generated features, severity-associated features were extracted and clustering was performed. Clustering results were compared for each variable type, and a better-performing clustering result was selected. By combining clusters and severity, subtypes were defined and their characteristics were identified.

To further elucidate this perspective, in this study, we tried to examine the molecular heterogeneity of COVID-19 patients by converting Cell-Cell Interaction (CCI) score, an inter-cellular feature, to pathway-level using GSVA algorithm to reduce sparsity of CCI score and then clustering based on enrichment score for Ligand-Receptor (LR) pair set (see Fig. 1).

Clustering based on CCI Gene Set Variation Analysis (GSVA) score performed better than clustering based on gene expression or pathway GSVA score. In addition, the Moderate group was divided into two clusters, and the clusters were subdivided according to severity and then the immunological character was compared through the known marker. In conclusion, we discovered a severe-like moderate subtype “C2M” whose molecular characteristics are similar to those of severe and whose clinical characteristics are similar to moderate. As a result of examining worsening patients, all Peripheral Blood Mononuclear Cell (PBMC) samples at the time immediately before exacerbation were classified as C2M. Focusing on this point, we searched for factors similar to or different from C1M and C2S, focusing on C2M, and found marinal markers between subtypes. These findings are expected to play an important role in the selection of stratification and patient-specific treatments in COVID-19 patients.

## Results

### Clustering evaluation

We selected features that showed significant differences between severe and moderate cases for each data type of CCI GSVA, Pseudobulk, and Pathway GSVA. We then used k-means clustering to analyze the selected features in each data type. The clustering results showed that all three data types had a maximum silhouette score when k was 2 (see Supplementary Fig.1 ,Table S4). In the Discovery dataset, we named a cluster containing a majority of the Severe group based on the World Health Organization Ordinal Scale (WOS) a severe-enriched cluster, and a cluster that did not contain a majority of the Severe group a Moderate-enriched cluster. We evaluated the clustering result for the three data types (CCI GSVA, Pseudobulk, and Pathway GSVA) in terms of reproducibility and severity-association.

In-Group Proportion (IGP) analysis was performed to evaluate the reproducibility of the clusters. IGP analysis assesses how robust and reliable the clusters discovered in the discovery set are when replicated on external datasets^8^. After computing the distance between the data points of the external dataset and the cluster centroids of the discovery dataset, the sample is assigned to the cluster of centroids with the minimum distance. For each external data point assigned to each cluster, calculate the ratio of how many nearest neighbors were assigned to the same cluster. This ratio, called IGP value, evaluates the cluster’s reproducibility.

Cluster results based on CCI GSVA showed better reproducibility than cluster results based on two different variable types. Clusters based on CCI GSVA had high IGP values of 0.9 or higher on both external datasets, but clusters based on Pathway GSVA and Pseudobulk showed low reproducibility on certain datasets or specific clusters (see Table 1).

Since features used for clustering each variable type (CCI GSVA, Pathway GSVA, and Pseudobulk) were selected based on severity, we can assume the cluster result is related to severity. We evaluated the severity association for the three cluster results through clustering purity for severity. In the discovery dataset, Pseudobulk and CCI GSVA-based clusters had the same purity value. However, in the external dataset, CCI GSVA had a higher purity. Based on these results, we found that the CCI GSVA-based clustering result had a higher reproduction rate and better reflected the severity (see Table 1).

**Figure 2:**
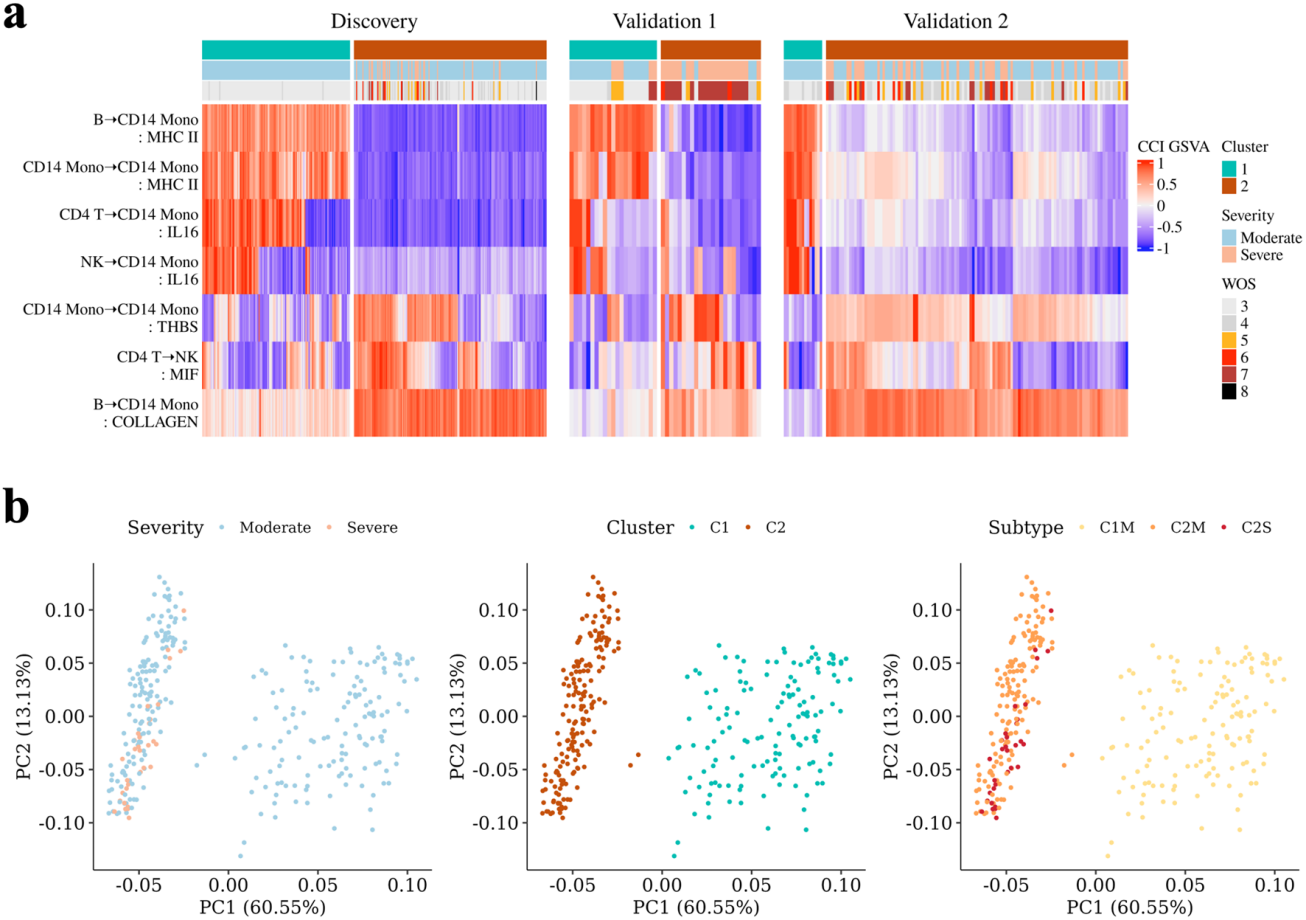
Clustering results. (**a**) Heatmap showing the clustering results for each dataset. (**b**) PCA results based on CCI GSVA in the Discovery set. Each point represents a sample, colored according to severity, cluster, and subtype.

The results of the CCI GSVA-based clusters are displayed in Fig. 2a as a heatmap. In the Discovery dataset, all of the samples in Cluster 1 were moderate, while Cluster 2 included all of the severe samples and some moderate samples. “MHC-II” interaction and “IL16” interaction were enriched in Cluster 1. Notably, “MHC-II” interactions were enriched in all samples belonging to Cluster 1. particularly for all Cluster1 samples of “MHC-II” interaction. Cluster 2 had upregulated LR pair sets that included “COLLAGEN” interaction from B to CD14 Monocyte, “THBS” signaling through autonomous interactions in CD14 monocytes, and “MIF” from CD4 T cell to NK. The “MHC-II” interaction in Monocyte was degraded, which is in line with the pattern observed in dysfunctional monocytes reported to increase in severe patients^9^. The tendency and pattern of the severity class distribution and enriched interaction observed in the discovery data set were consistent with those in the validation data set.

### Characterization of CCI-based subtypes

To explore the correlation between severity and clusters in COVID-19, we divided Cluster 2 into two groups: moderate (C2M) and severe (C2S), based on the level of severity. As illustrated in Fig. 2, C2M was more closely related to the samples in the severe group than to the samples in Cluster 1, which belonged to the same moderate group.

In order to confirm that the moderates of Cluster 2 (C2M) are close to severe only in CCI GSVA, the difference between each subtype was examined using the already known severity related biomarkers (Known markers) in the COVID-19 single cell transcriptome study.

**Figure 3:**
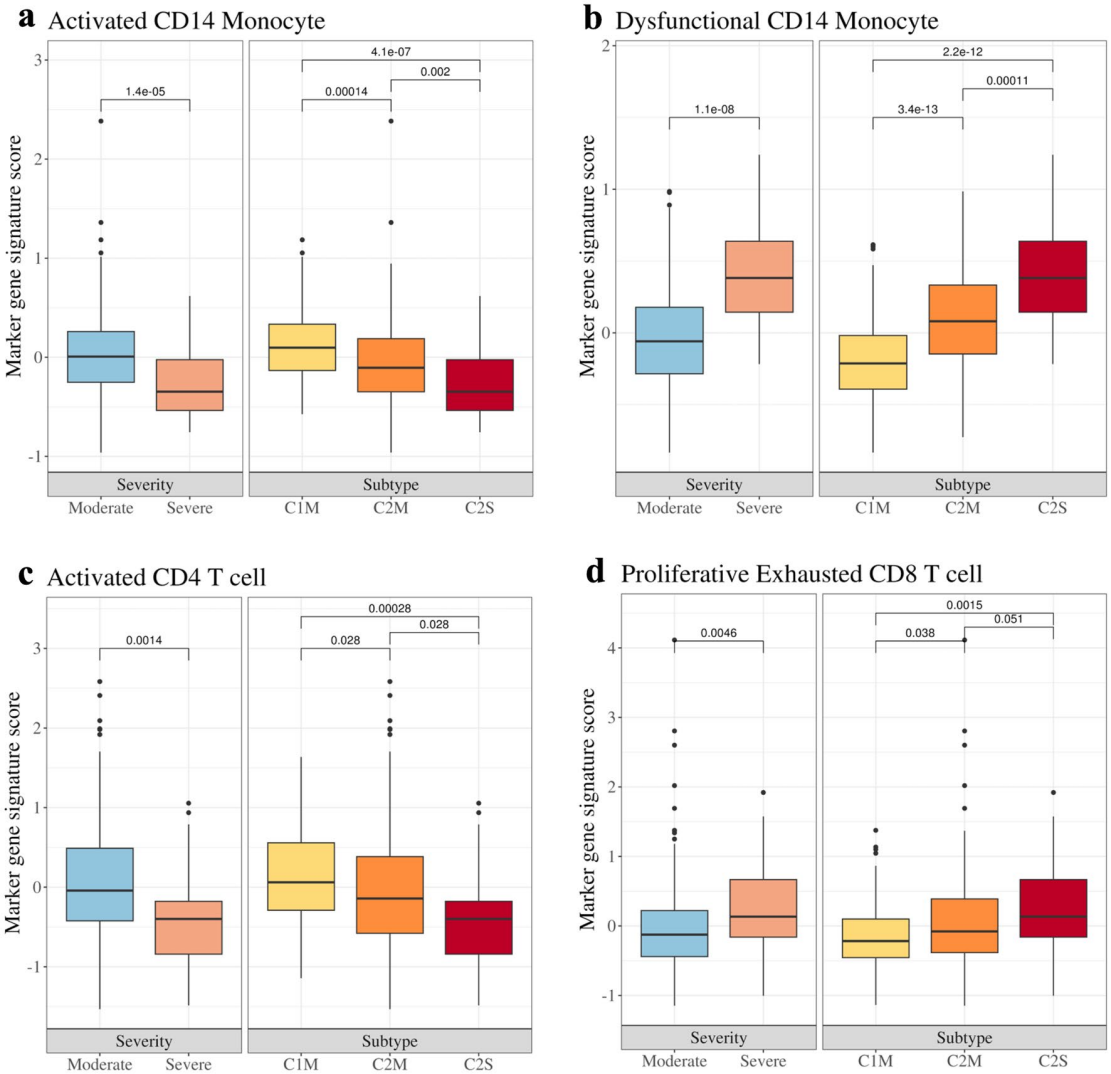
Boxplots for subtypes and markers (cell types) associated with COVID-19 severity. Gene signature scoring was conducted for marker gene sets of cell types known to be associated with severity.

Intermediate pattern of C2M subtype between C1M and C2S was seen in marker gene signature score of activated monocytes and dysfunctional monocytes, known as severity-related subpopulation^9^. We selected 2 types of cell subpopulation in CD14 monocyte known to be related with severity (see Fig. 3a,b). Activated monocytes, which is represented by high expression of *HLA-DR* and *CD83*, are commonly found in moderate patients, while dysfunctional monocytes, which express low *HLA-DR* and high alarmins (*S100A8/9/12*), exist in high level in severe patients compared to the moderate ones^9^. In our data, marker gene signature score of activated monocytes exhibited lower expression in moderate samples relative to severe samples, as expected. Our new found subtype, C2M showed an intermediary expression level between C1M and C2S. In contrast, severe samples were highly above moderate samples by marker gene signature score of dysfunctional CD14 monocytes. Similar to the previous description, scores of C2M samples were in-between C1M and C2S samples.

In addition, there are other COVID-19 related factors expressed in CD4+ T cells (see Fig. 3c). *CD2AP* expressed on the surface of CD4+ T cells regulates follicular helper T cell differentiation and enhances antibody responses during viral infection. *TNFSF14* activates and recruits T cells from peripheral blood to tissues. Altogether, these markers are T cell activation promoting factors, and are known to be elevated in COVID-19 patients compared with healthy controls^10^. In our data, severe patients showed decreased expression of these markers, implying that reduced T cell activation, recruitment, antibody responses might have resulted in severe conditions. C2M exhibited intermediate expression between C1M and C2S as well.

Lastly, some genes expressed in CD8+ T cells are identified as markers for proliferative exhausted CD8+ T cell, which shows positive correlation with COVID-19 severity(see Fig. 3d)^11,12^. These signatures displayed higher expression in severe population of our data as well. C2M demonstrated transitional state as same as the other known markers, but was slightly closer to C2S.

In conclusion, since C2M subtype exhibited similar immunophenotype to C2S while clinical phenotype characterized it as moderate, we defined C2M subtype as “severe-like moderate”.

### Association analysis between subtype and clinical trait

**Figure 4:**
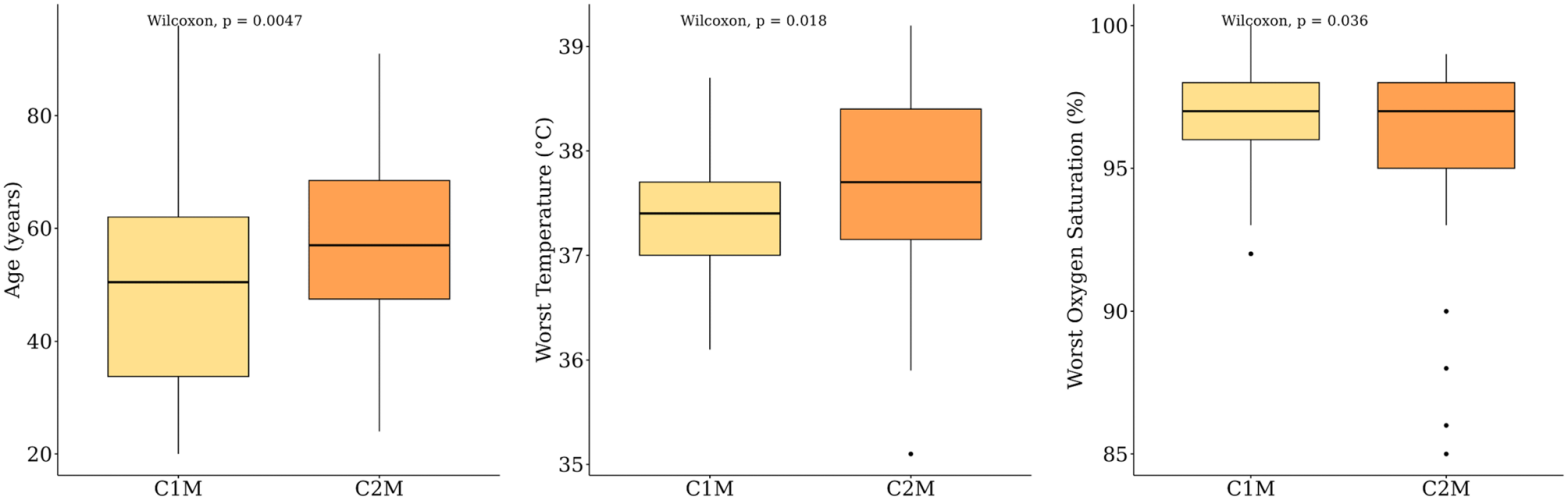
Boxplot for continuous clinical variables with significant differences between C2M and C1M.

This analysis was conducted to investigate the clinical variables associated with the differences between the C2M and C1M groups, both of which presented with moderate symptoms. As the clusters were designated based on samples, patients were assigned to clusters according to their earliest available sample. The analysis was carried out separately for both continuous and categorical variables (see Fig. 4, Supplementary Table S2, S3).

Among the continuous clinical variables, significant differences were found between the subtypes in age, body temperature (the worst recorded during hospitalization), and oxygen saturation (the worst recorded during hospitalization). Age and body temperature (worst recorded) were significantly higher in C2M compared to C1M, while oxygen saturation (worst recorded) was significantly lower in C2M.

Regarding the categorical clinical variables, significant differences were observed in diabetes (comorbidity), low-dose oxygen therapy via nasal prong during hospitalization, ECOG performance status at the time of admission, and presence of pneumonia on chest X-ray/CT scan at the time of visit. Specifically, among the 55 patients in C2M, 18 had diabetes, while of the 80 patients in C1M, 9 had diabetes as a comorbidity. The proportion of patients with limited physical activity according to ECOG performance status was significantly higher in C2M. In the Severe group, the 23 samples were obtained from 7 male patients, while the 9 samples were from 3 female patients, indicating a gender bias. However, in the C1M and C2M groups, the gender distribution was more balanced with 42 males and 38 females in the C1M group, and 28 males and 27 females in the C2M group. Fisher’s exact test confirmed that this distribution was not significantly different (p.value = 0.863).

Mostly, between C1M and C2S, C2M also showed significant differences with C2S, presenting intermediate characteristics, but there were clinical variables showing distinguishing patterns. In the case of the worst body temperature, C2M had a distinctively higher value compared to the other two subtypes (see Supplementary Figure 2). Regarding the findings of pneumonia, there was no significant difference between C2M and C2S. Half of the patients in C1M (40/80), 73% of the patients in C2M (40/55) had pneumonia findings at the time of visit, and all patients in C2S had pneumonia findings at the time of visit (see Supplementary Table S5). The nasal prong low-dose oxygen therapy showed no difference between Moderate and Severe, as the Moderate had fewer cases receiving oxygen therapy, and Severe patients received a higher level of oxygen therapy like facial mask, therefore, there was no frequency difference observed.

From these observations, it could be inferred that C2M had intermediate characteristics between Moderate (C1M) and Severe in terms of age, ECOG performance, and comorbid diabetes. A distinctive feature of C2M was the occurrence of high fever symptoms. Moreover, it was observed that the probability of belonging to C2 (C2M, C2S) was higher in cases with pneumonia findings.

### Longitudinal analysis

**Figure 5:**
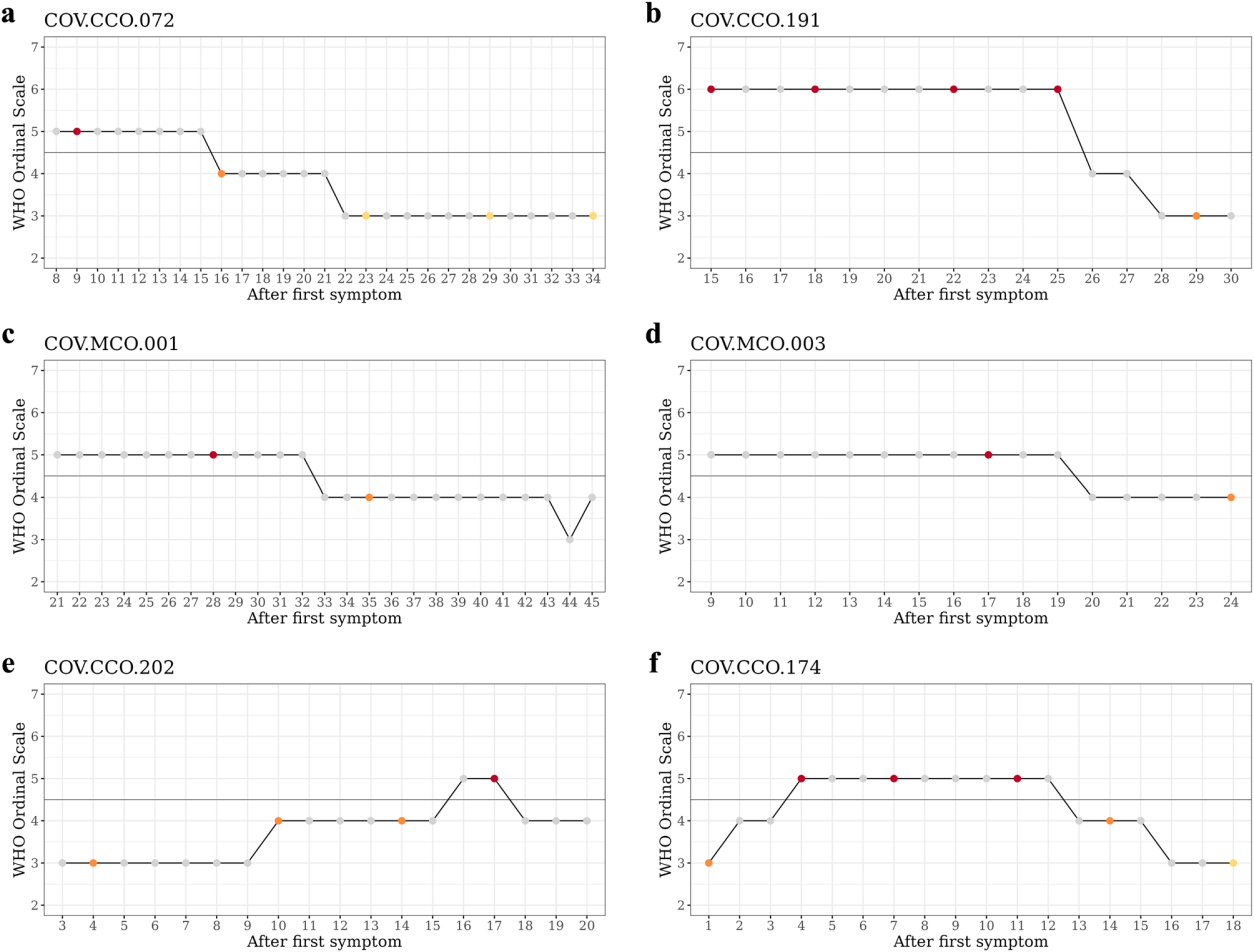
Line plot illustrating the severity (WOS) and subtype at each timepoint for patients with worsened or alleviated conditions. (**a**–**d**) represent patients who experienced alleviation, while (**e** and **f**) depict patients who initially worsened and subsequently improved. the color of the dots represents the severity of the patient’s condition and whether single cell RNA-seq was used at that time point. Red indicates severe conditions, orange indicates moderate conditions, and grey is used when single cell RNA-seq was either not available.

We identified subtypes and explored their associations with the patterns of worsening/improvement in patients who experienced a change in severity from moderate to severe or from severe to moderate (see Fig. 5, Supplementary Table S2). We selected patients with worsening changes from the discovery dataset (2 patients), validation dataset 1 (1 patient), and validation dataset 2 (2 patients). Among these 5 patients, 4 patients’ samples were classified as C2M before worsening to severe. When only PBMC samples were considered, all samples before worsening from moderate to severe were classified as C2M. Conversely, in patients who improved from severe to mild, their molecular characteristics remained similar to those of Cluster 2 despite the changes in severity. In the case of COV.CCO.072, the sample changed to C1M seven days after improvement to moderate.

These findings suggest that the molecular subtype is related to the patterns of worsening or improvement in COVID-19 patients. Patients who worsened to severe tended to belong to C2M before the worsening, while patients who improved to moderate tended to maintain the characteristics of Cluster 2 (C2M) even after their improvement. These results may provide insights into the underlying mechanisms of COVID-19 severity and help in developing targeted therapies based on molecular subtypes.

### Marginal markers between subtypes

In this part of our study, we set out to identify the subtle and intermediate differences among the C2M, C1M, and C2S groups. Even though patients in C2M presented with moderate symptoms, they found themselves clustered with the severe symptom group (C2S) due to similar intercellular features. This ambivalence in their classification hinted at an underlying complexity, prompting us to investigate further.

**Figure 6:**
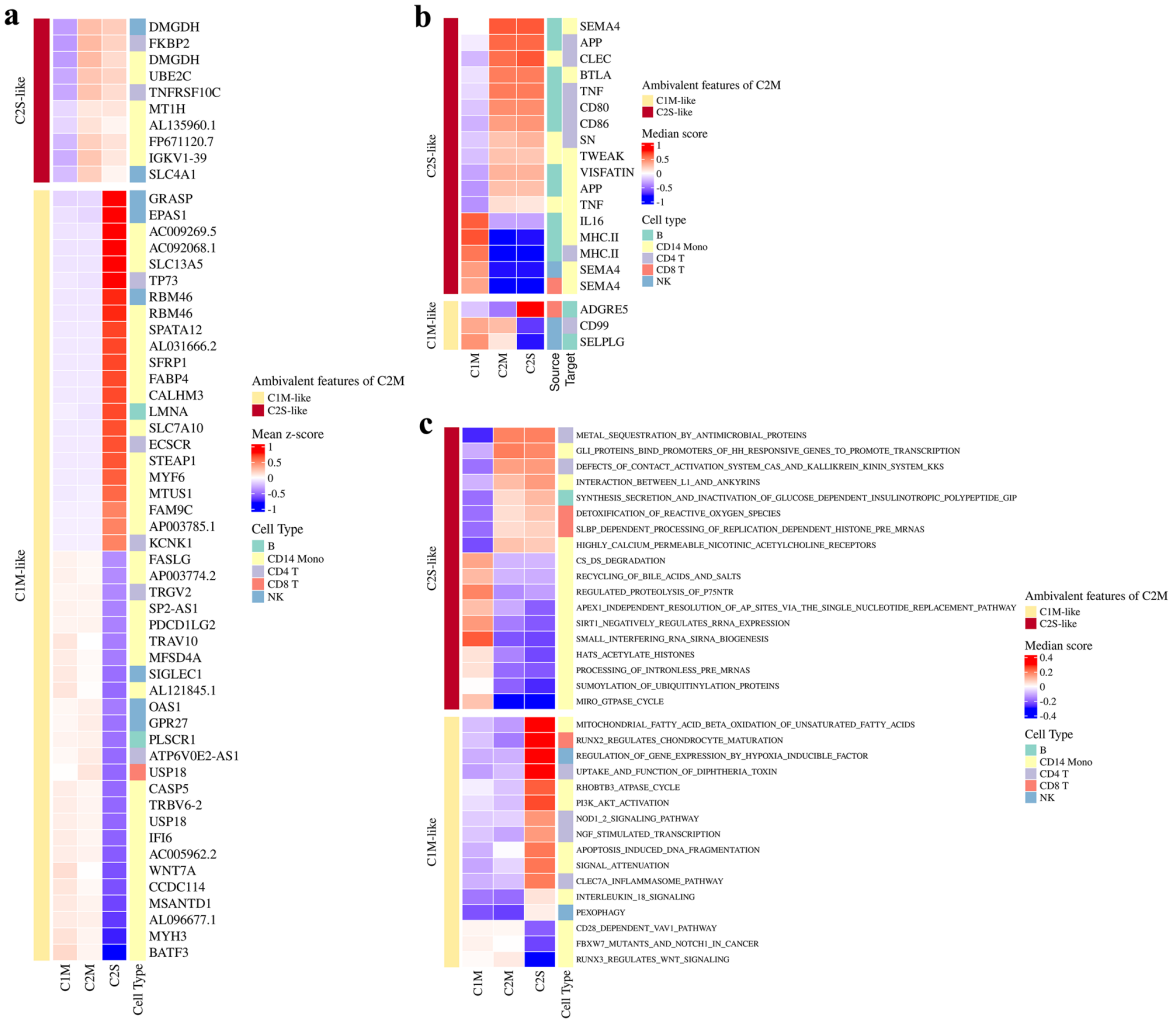
Heatmap of marginal markers to visualize the dual characteristics of C2M. Factors exhibiting a similar pattern to C2S are defined as C2S-like, and those showing a pattern similar to C1M are defined as C1M-like. (**a**). Results from gene analysis, (**b**). Results from CCI GSVA, (**c**). Results from pathway GSVA.

By arranging the variables using C2M as a base, we hoped to uncover specific marginal markers that could shed light on this ambivalence. These markers helped us highlight the intermediate differences between C2M and the other two groups. We coined the terms “C1M-like” and “C2S-like” to categorize these markers. Factors that diverged between C2M and C2S but were similar to C1M were designated “C1M-like”. Conversely, factors that differed between C2M and C1M but were akin to C2S were labeled “C2S-like” (see Fig. 6).

We assumed that by dissecting the ambivalence within C2M, we could identify markers instrumental in determining clinical severity. This study sets the groundwork for such investigations, enhancing our understanding of how these patient clusters are determined and offering insights into the nature of disease progression.

#### C1M-like factors

Several markers included in C1M-like factors have notable functions related to COVID-19 severity, which could help elucidate the identity of C2M subtype. *PLSCR1*, induced by interferon, is known for playing a crucial role in enhancing the antiviral response through increased expression of a select subset of potent antiviral genes^13^. Similarly, *OAS1* is also an IFN-responsive gene, which can contribute to innate antivral response in cells. Specifically, C-terminal prenylated *OAS1* can bind to SARS-CoV-2 5’UTR in membranous replicative oragnelles of SARS-CoV-2 infected cells, and block virus replication^14^. While these markers were upregulated compared to C2S, there are other C1M-like marginal markers suppressed in C1M and C2M. *EPAS1*, which is called as HIF-2alpha as well, is induced by hypoxia, playing a key role in virus infection and pro-inflammatory response. COVID-19 patients exhibit dysregulated HIF-1alpha signaling, showing high expression, which leads to immune-inflammatory cytokine expression^15^.

Several pathways associated with COVID-19 severity were identified as C1M-like factors. Pathways related to “Mitochondrial fatty acid beta oxidation of unsaturated fatty acids”, “PI3K AKT activation”, and “IL-18 signaling” were identified in CD14 monocytes, while the “Regulation of gene expression by hypoxia inducible factor” was identified in NK cells, and “NGF sitmulated transcription” was identified in CD4 T cells. In particular, IL-18 signaling in the lung has been reported to activate inflammasomes associated with disease severity, and levels of IL-18 have been observed to increase over time in deceased patients^7^. These pathways have been previously linked to COVID-19 severity and prognosis, with mitochondrial fatty acid beta oxidation, nerve growth factor, and hypoxia inducible factor showing particular relevance^16–19^.

#### C2S-like factors

On the other hand, C2S-like factors were also detected in our research, and could have a possibility of interpreting the phenomenon of COVID-19 severity. *TNFRSF10C* is part of the TNF-receptor superfamily, comprising a transmembrane domain and extracellular TRAIL-binding domain. TNFRSF members control innate and adaptive immune cells by co-stimulating or co-inhibiting immune responses. High expression of TNF-related genes is observed in severe conditions and is associated with mortality^20^.

In CCI analysis, TNF signaling between B cells and CD4 T cells was identified as a C2S like factor. TNF-related genes identified through gene analysis were found to affect the interaction between these cell pairs.

Pathway analysis revealed pathways previously known to be related to COVID-19 severity. The “Metal sequestration by antimicrobial proteins” pathway was identified in CD4 T cells, and the presence of metal ions at various concentrations in hospitalized COVID-19 patients has already been reported^21^. The Kallikrein kinin system pathway was also identified, which is known to be related to inflammation, vascular homeostasis, and coagulation in COVID-19 pathology^22^. The study also reported a relationship between the activation of the Kallikrein kinin system and the prognosis of COVID-19 patients. The “Detoxification of reactive oxygen species” pathway was identified in CD8 T cells, as tissue damage, thrombosis, and red blood cell dysfunction can be induced by reactive oxygen species in COVID-19 patients^23^. However, this study focused on the relationship with neutrophils. In CD14 monocytes, pathways related to highly calcium permeable nicotinic acetylcholine receptors, recycling of bile acids and salts, and SIRT negatively regulating rRNA expression were identified. Nicotinic acetylcholine receptors (nAChRs) are known to affect cytokine liberation and virus replication. The association between increased serum levels of bile acids and inflammation has been reported, and it has been suggested that this is related to tissue damage in COVID-19 disease progression^24,25^. There is also a study reporting changes in bile acid metabolism in COVID-19 hospitalized patients^26,27^. *SIRT-1* is related to NAD+ levels, which are known to decrease with age, hypertension, obesity, and diabetes. There is a study reporting a decrease in NAD+ levels in patients infected with COVID-19^28^. In addition, it has been suggested that T and B cell signaling and lymphocyte homeostasis may also be affected.

### Evaluation of subtype predictivity using machine learning

We could not identify any specific markers that were up- or down-regulated in the C2M subtype among the variables such as Gene, Pathway, and CCI. Based on the known markers, the C2M subtype exhibited intermediate characteristics compared to other subtypes. Therefore, we constructed a logistic regression model to verify whether C2M could differentiate from other subtypes. Additionally, we evaluated whether our devised subtype classification criteria, which was based on molecular characteristics, was a better classification criterion than the severity-based moderate/severe classification by constructing a logistic regression model for the severity class independently and comparing the classification performance of both models (see Table 2).

Although the severity classification was binary, and the subtype classification was multi-class, the performance of the multi-class subtype classification model outperformed the severity classification model. Although the classification performance for severe cases was low, we observed an improvement in the classification performance for severe cases (C2S) after classifying moderate cases into C1M and C2M.

These results suggest that our newly defined subtypes based on molecular features could provide additional information beyond the conventional severity classification, and highlight the potential value of integrating molecular and clinical features for improved patient stratification and personalized treatment in this disease.

### C2M specific cytokine markers

**Figure 7:**
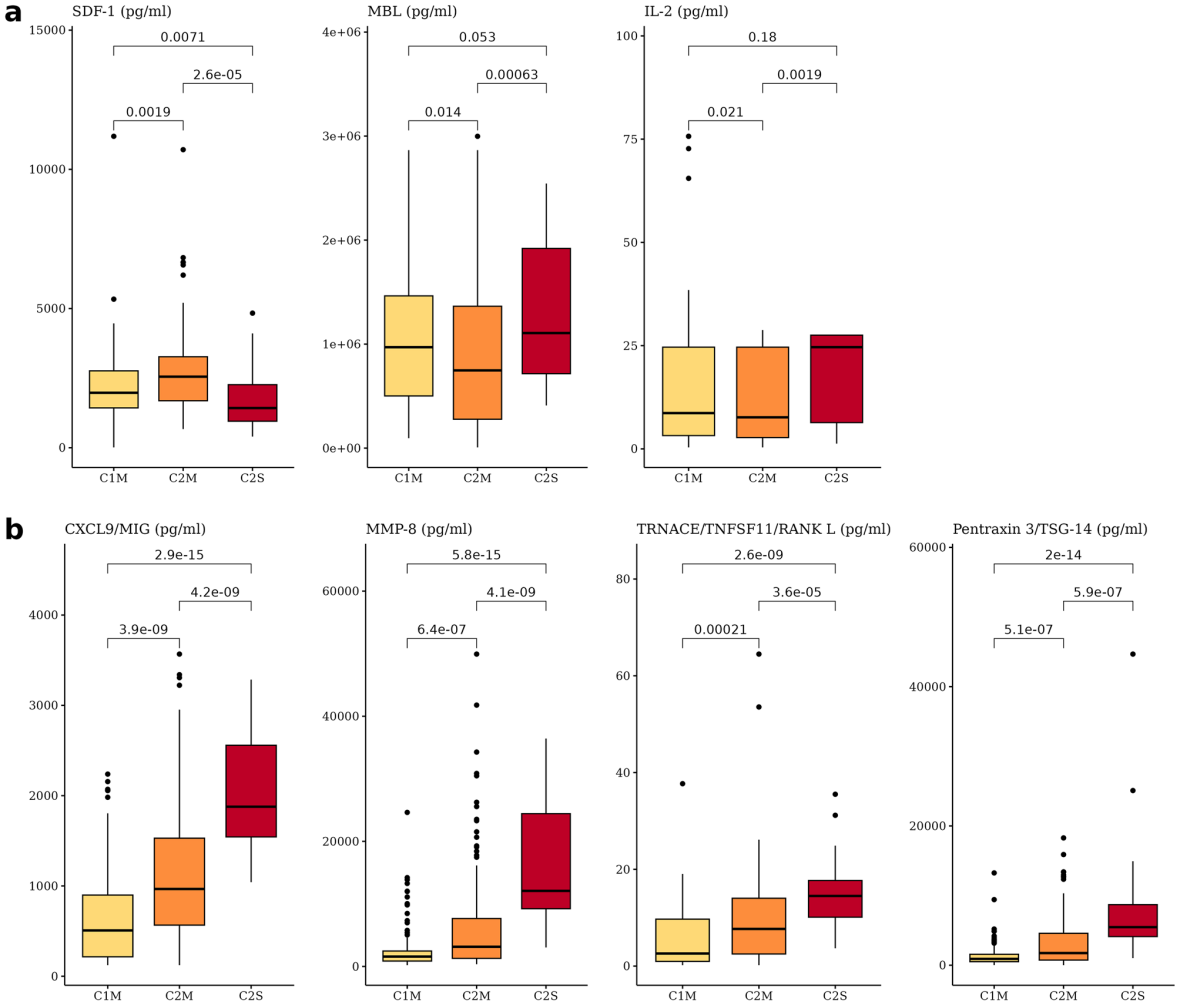
Boxplots for C2M-specific cytokines. (**a**) Cytokine markers specifically up- or down-regulated in C2M. (**b**) Cytokine markers with significant differences between C1M and C2M.

To identify specific markers of C2M, we explored cytokines and conducted Wilcoxon rank-sum tests to determine differences between groups. Significant differences were defined as those with an FDR (False Discovery Rate) < 0.05. Among the cytokines showing significant differences with other groups and with the lowest or highest median of C2M, SDF-1, MBL, and IL-2 were selected as potential markers (see Fig. 7a).

The analysis revealed that SDF-1 was up-regulated in C2M, while it showed a low expression trend in the severe group (C2S). SDF-1 is a cytokine known to promote the movement of hematopoietic stem cells and immune cell infiltration to infection sites. The up-regulation of SDF-1 in COVID-19 patients with C2M may be interpreted as a host response to the virus, which leads to the migration of a large number of immune cells to the infection site. This migration of immune cells could potentially accelerate viral clearance at the site of infection.

In contrast, MBL and IL-2 showed decreased expression in patients with moderate COVID-19 symptoms (C2M), while they exhibited higher expression in the severe subtype (C2S). Both cytokines were up-regulated in C2S, indicating a stronger immune response in severe cases. MBL is an important protein of the complement system that plays a crucial role in viral clearance. IL-2 is a cytokine that regulates the proliferation and activation of T cells, which is closely related to the immune response^29^. Further studies have implied that IL-2 could be a key factor in predicting neutralization levels, particularly emphasizing its role in T cell activation and proliferation^7^. Therefore, the increase in MBL and IL-2 expression in severe COVID-19 patients (C2S) can be interpreted as an attempt of the immune system to fight off the virus. On the other hand, compared to the other subtypes, it can be interpreted that this viral response is relatively less pronounced in C2M.

In patients with C2M, the expression of SDF-1 was up-regulated, indicating a host response to the virus and potentially accelerating viral clearance at the infection site^30^. However, the expression of MBL and IL-2 was decreased in C2M, suggesting a less pronounced viral response compared to other subtypes.

The findings suggest that the expression levels of SDF-1, MBL, and IL-2 could be useful biomarkers for stratifying COVID-19 patients into different subtypes. Monitoring the expression of these cytokines in COVID-19 patients could provide valuable information for predicting disease progression and identifying patients who may benefit from more aggressive treatment or interventions. Therefore, the results of this study could have important clinical implications for the management of COVID-19 patients.

We identified cytokines that showed significant differences even within the moderate subtypes (see Fig. 7b). Among C1M and C2M, which were both classified as moderate, we found differences in CXCL9/MIG, MMP-8, TRANCE/TNFSF11/RANK L, and Pentraxin3/TSG-14. We used an absolute relative median difference > 0.45 and a Wilcoxon rank-sum test FDR < 0.01 as the criteria for determining significant differences. Interestingly, even among the moderate subgroups, there were significant differences in cytokine expression. CXCL9/MIG, MMP-8, TRANCE/TNFSF11/RANK L, and Pentraxin3/TSG-14 showed differences between C1M and C2M, with C2M having intermediate levels between the moderate and severe groups.

CXCL9/MIG plays a role in recruiting immune cells to sites of infection and inflammation^31^. The higher levels in severe cases suggest a stronger immune response that could help control the progression of the disease. MMP-8 is involved in tissue repair and remodeling and can also be produced by immune cells in response to infection^32^. Higher levels in severe cases could reflect increased tissue damage and inflammation. TRANCE/TNFSF11/RANK L is involved in bone remodeling and can activate immune cells^33^. Higher levels in severe cases could reflect the involvement of bone tissue in the disease process or an increased need for immune activation. Pentraxin3/TSG-14 is involved in the innate immune response and can bind to microorganisms and other molecules to help clear infections^34^. Higher levels in severe cases could reflect a more robust immune response to the virus.

The findings suggest the presence of molecular subtypes that have not been clinically classified, which could have implications for understanding the mechanisms underlying COVID-19 and developing targeted therapies.

## Discussion

In this study, we conducted a comprehensive clustering analysis on 320 COVID-19 PBMC samples using single-cell RNA-seq data. Our analysis included diverse demographic and clinical characteristics, ensuring robust and representative results. The validity of our findings was further reinforced by performing similar analyses on external datasets, which confirmed the existence and characteristics of the subtypes we discovered.

Our approach utilized inter-cellular feature-based CCI GSVA, which allowed for the creation of severity-associated clusters with higher reproducibility than intra-cellular feature-based methods. This finding underscores the critical role of cell-cell interactions in the immune response and the severity of COVID-19. Interestingly, no cluster consisting exclusively of moderate cases was found except in the case of CCI GSVA, suggesting the importance of inter-cellular communication in disease progression.

Interestingly, we observed significant differences in known severity-associated markers not only between severity groups but also between C1M and C2M, both of which are classified as moderate. This implies that traditional clinical severity classifications may be insufficient in capturing the nuanced immune responses at the molecular level, even within the same severity level. These findings underscore the need for the establishment of molecular subtypes for more precise patient stratification and individualized treatment strategies in infectious diseases such as COVID-19. Our study also raises questions about the current clinical guidelines that rely heavily on severity labels, advocating for a more nuanced approach that incorporates molecular data into patient management.

Despite some limitations in our study, such as not using a specialized algorithm for CCI and the use of an LR pair set database, and the focus being on inter-sample heterogeneity within given severity levels rather than transitions between different severity levels, our work provides valuable insights into the molecular mechanisms underlying the pathogenesis of COVID-19. This could facilitate the development of targeted therapies based on patient-specific molecular subtypes, and pave the way for more detailed patient stratification in the future.

Future studies could extend the scope of this work by investigating patient heterogeneity in more depth, possibly incorporating transitions from moderate to severe and vice versa based on molecular features. Moreover, this methodology offers a scalable framework that can be adapted for studying other infectious diseases, thereby broadening its applicability beyond COVID-19. Our findings also contribute to the existing body of literature by offering a more nuanced understanding of severity-associated molecular features, thereby filling a critical gap in current research that often overlooks intra-group heterogeneity.

## Methods

### Used data

A total of three datasets were used in this study (see Table 3). The Discovery set consists of 320 peripheral blood mononuclear cell (PBMC) samples derived from 145 COVID-19 patients^35^. Validation set 1 comprises 45 samples from 25 patients, while validation set 2 is composed of 133 samples derived from 79 patients^9,12^. All samples were collected when the patients’ severity was at a WHO ordinal scale of 3 or higher.

The Discovery set, which contains the largest number of samples, was used to derive clusters, evaluate clustering, and perform all downstream analyses. Validation sets 1 and 2 were employed for selecting severity-associated features and calculating the reproducibility and clustering purity of the derived clusters. These sets were also used to compare the clustering performance according to different variable types (CCI GSVA, pathway GSVA, and pseudobulk gene expression).

### Preprocessing single cell RNA sequencing data

We followed COVID-19 PBMC single cell RNA-sequencing preprocessing method proposed by^36^, but applied a more strict quality control metric. Each single cell RNA-seq sample was preprocessed using Seurat^37^. Cells that did not meet the following criteria were filtered out: the number of expressed genes in a cell should be greater than 200, and the proportion of mitochondrial gene expression counts to the total expression counts should be less than 50%. Cell type annotation was performed using PBMC reference single cell dataset and data transfer method implemented in Seurat, and cells with mapping, prediction score exceeding 0.6 were left^38^. CD14 Monocyte, CD16 Monocyte labelled cells were filtered using prediction score of level 2, and level 1 prediction score for the remaining cell types. Also, genes expressed in more than 5 cells were left.

To select cell types found in most of the patients, and LR pair interactions frequently detected among them, cell types with more than 10 cells in at least 90% of all samples were selected, and we used samples possessing more than 10 cells of the selected cell types.

### Cell-cell interaction enrichment analysis

Cell-cell interaction scores (CCI) were calculated individually for each sample using *CellChat*^39^. The Ligand-Receptor (LR) pair level CCI was calculated through the basic procedure presented by *CellChat*.

In the database *CellChatDB.Human*, each LR-pair corresponds to a gene, and the pathway information to which the LR pair belongs is corresponding to a gene set. CCI pathway score (CCI GSVA) was calculated for each source-target cell type pair. *gsva* function of the R package *GSVA* was used to calculate the engineering score, and Gaussian kernel was used as kernel function^40^.

### Pathway enrichment analysis

Pathway enrichment analysis was performed for each cell type in the sample for pseudobulk expression. The Gaussian kernel was used using function *gsva* of the *GSVA*. KEGG, REACTOME, and GO of msigdb v7.5.1 were used gene sets^41,42^.

### Severity associated feature selection

Features showing significant differences between severe and moderate were selected in all three datasets (Discovery, Validation 1,2). In the case of gene expression, the FDR in the DEG test was less than 0.05, and for CCI and pathway, t-test was conducted on the difference in enrichment score between the two groups. Pathways (gene set, CCI set) with p.value less than 0.05 were defined as severity associated feature. In all datasets, only variables with the same sign of difference between the severe and moderate were selected.

### Clustering analysis

K-means clustering algorithm was used. After performing all of parameter k=2,3,4, and 5, k having the maximum average silhouette score was selected. The *kmeans* function of the R package *stats* was used, and clustering was performed by setting the parameter to maximum iteration = 200 and the number of start points = 50. In the case of pseudobulk gene expression, k-means clustering was performed after scaling with z-score for the aggregated gene count of each cell type. Using function *IGP.clusterRepro* in the R package *clusterRepro*, clustering reproductivity was evaluated on two validation sets^8^. After calculating the euclidean distance between samples in the validation set and centroid in the discovery set, samples were assigned to a cluster of centroid with the minimum distance. The following equation (1) was used to calculate the clustering purity.

1 $$\begin{aligned} \text {Purity}\,(\mathcalligra {C}, \mathcalligra {S}) = \sum _{i=1}^{k_c} w_i \cdot \max _{j=1}^{k_s} \frac{\text {count}(c_i, s_j)}{\text {count}(c_i)} \end{aligned}$$

In this equation, the variables represent the following:

- $$\mathcalligra {C}$$: Cluster labels
- $\mathcalligra {S}$: Severity class labels
- $k_c$: Maximum number of cluster labels
- $k_s$: Maximum number of severity class labels
- $w_i$: Weight of cluster *i* (the proportion of data in cluster *i* relative to the total data)
- $\text {count}(c_i, s_j)$: The number of data points with cluster label *i* and severity class label *j* occurring simultaneously

### Gene signature scoring

For cluster description, we compared gene signature scores constructed by known markers related to COVID-19 between clusters. Gene signature score was generated by calculating the mean z-score of selected genes. In the case of activated, dysfunctional monocytes, we selected top 30 marker genes per cell type based on average log2-transformed fold change.

### Association analysis between subtypes and clinical traits

To investigate the association between subtype and clinical trait, we conducted association analysis. For categorical variables, Fisher’s exact test was performed, and for continuous variables, Wilcoxon rank-sum test was conducted. A p-value less than 0.05 was considered statistically significant.

### Marginal marker analysis

We conducted a marginal marker analysis to identify variables that are differentially expressed between two groups. First, we divided the variables based on the C2M subtype classification. We defined C1M-like variables as those that show a significant difference between C2M and C2S, but not between C2M and C1M. Conversely, we defined C2S-like variables as those that show a significant difference between C2M and C1M, but not between C2M and C2S. To identify these variables, we applied the following criteria: absolute median difference> 0.4 and FDR< 0.01 with opposite sign of median for CCI GSVA values, and absolute median difference> 0.2 and FDR< 0.01 with opposite sign of median for pathway GSVA values. We also considered variables with p-values greater than 0.5 as non-significant or “same”. We used t-tests to perform statistical testing. In case of genes, we performed pairwise DEG analysis between each subtype via edgeR and employed PValue, FDR, logFC values to determine marginal markers. Following criteria were used: absolute value of logFC$\ge$ 1.0 and FDR< 0.01 as significantly different variables, and absolute value of logFC< 0.01 and P.value> 0.9 as non-significant variables.

### Evaluation of subtype predictivity using machine learning

To validate the effectiveness of the new types of cluster labels proposed in this paper, a simple classification model using the CCI GSVA score was used. The classification accuracy was measured using a multi nominal linear regression classifier with 3-fold cross validation with a linear regression model from python library *scikit-learn*. To build the logistic regression model, values of CCI GSVA scores were used as input value. In the case of class imbalance in the data, F1-score were used. Specifically, the macro average calculates each metric (F1, Recall, Precision) for each label independently, then takes the unweighted average of these scores, treating all classes equally. On the other hand, the weighted average gives more importance to the larger classes by weighting each metric (F1, Recall, Precision) by the number of samples from that class.

**Table 1:** Comparison of clustering performance based on different variable types. For each clustering result, In-group Proportion (IGP) analysis and severity purity calculations were conducted.

	Dataset	Cluster	CCI GSVA	Pseudobulk	Pathway GSVA
In-group proportion (IGP value)	Validation 1	Moderate enriched cluster	**1.00**	0.86	0.95
		Severe enriched cluster	**0.96**	0.87	0.83
	Validation 2	Moderate enriched cluster	**0.93**	0.91	0.88
		Severe enriched cluster	**1.00**	0.95	0.87
Clustering purity	Discovery		**0.79**	0.78	0.67
	Validation 1		**0.80**	0.65	0.74
	Validation 2		**0.98**	0.57	0.58

For IGP values, the calculation was performed after classifying clusters into moderate-enriched and severe-enriched clusters.

Significant values are in bold.

**Table 2:** Classification performance of logistic regression models for subtype and severity, respectively.

Classification	Class	Precision	Recall	F1-score
Subtype	C1M	0.986	1.000	0.993
	C2M	0.915	0.940	0.927
	C2S	0.731	0.594	0.655
	Macro average	0.877	0.844	0.858
	Weighted average	0.927	0.931	0.929
Severity	Moderate	0.945	0.962	0.954
	Severe	0.593	0.500	0.542
	Macro average	0.769	0.731	0.748
	Weighted average	0.910	0.916	0.912

**Table 3:** Sample and patient information for the single-cell RNA-seq datasets used in this study.

		Discovery set^35^	Validation set 1^9^	Validation set 2^12^
Total	# of samples (# of patients)	320 (145)	45 (25)	133 (79)
	Male	174 (77)	21 (13)	78 (45)
	Female	146 (68)	24 (12)	55 (34)
	Median age	58 (mean: 57.23, min: 20, max: 96)	63 (mean: 60.67, min: 23, max: 83)	64 (mean 62.75, min: 26, max: 89)
Moderate WOS < 5	# of samples	288	20	92
	Male	151	10	46
	Female	137	10	46
	Median age	57 (mean: 55.9, min: 20, max: 96)	63 (mean: 55.5, min: 23, max: 83)	68 (mean: 64.71, min: 26, max: 89)
Severe WOS > 4	# of samples	32	25	41
	Male	23	11	32
	Female	9	14	9
	Median age	68 (mean: 69.16, min: 49, max: 91)	63 (mean: 64.8, min: 43, max: 78)	61 (mean: 58.37, min: 33, max: 88)

### Supplementary Information

Supplementary Figures.

Supplementary Tables.

## Data Availability

The discovery dataset used for clustering in this study, including clinical data, cytokine profiling, and scRNA-seq, is available from the Clinical and Omics Data Archive (CODA; https://coda.nih.go.kr). The clinical data, cytokine profiling, and scRNA-seq datasets have been deposited at CODA with the accession numbers CODA-000034, CODA-000035, and CODA-000041, respectively. The COVID-19 single cell RNA validation sets are available at: https://github.com/RGLab/covid19_sc. Custom scripts for data analysis are available upon request. For further data requests, please contact the first author, Kyeonghun Jeong, at scientist0205@snu.ac.kr.
