# Supplementary material for: Subtyping of COVID-19 samples based on cell-cell interaction in single cell transcriptomes
Source: Sci Rep. 2023 Nov 10;13:19629. doi: 10.1038/s41598-023-46350-2 (PMC10638268; doi:10.1038/s41598-023-46350-2)
Supplement: Supplementary file 1 — Supplementary Figures. [file 41598_2023_46350_MOESM1_ESM.docx]

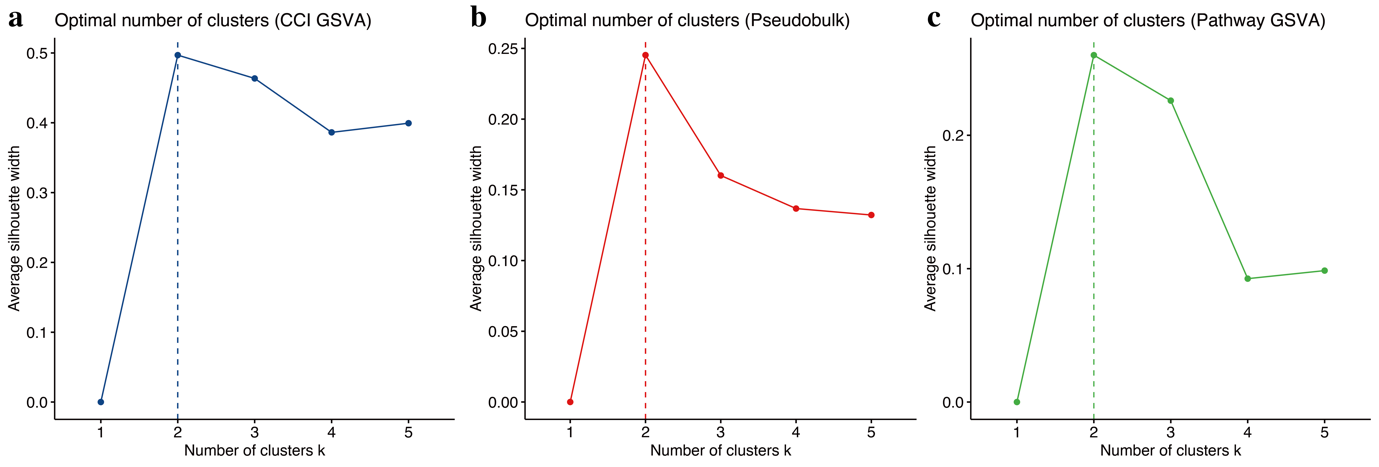


Supplementary Figure 1. Comparison of the average silhouette width for determining the optimal number of clusters for three different data types. a. CCI GSVA shows the maximum silhouette score at k=2. b. Pseudobulk also exhibits the highest score at k=2. c. Pathway GSVA demonstrates the same trend with the highest silhouette score at k=2.


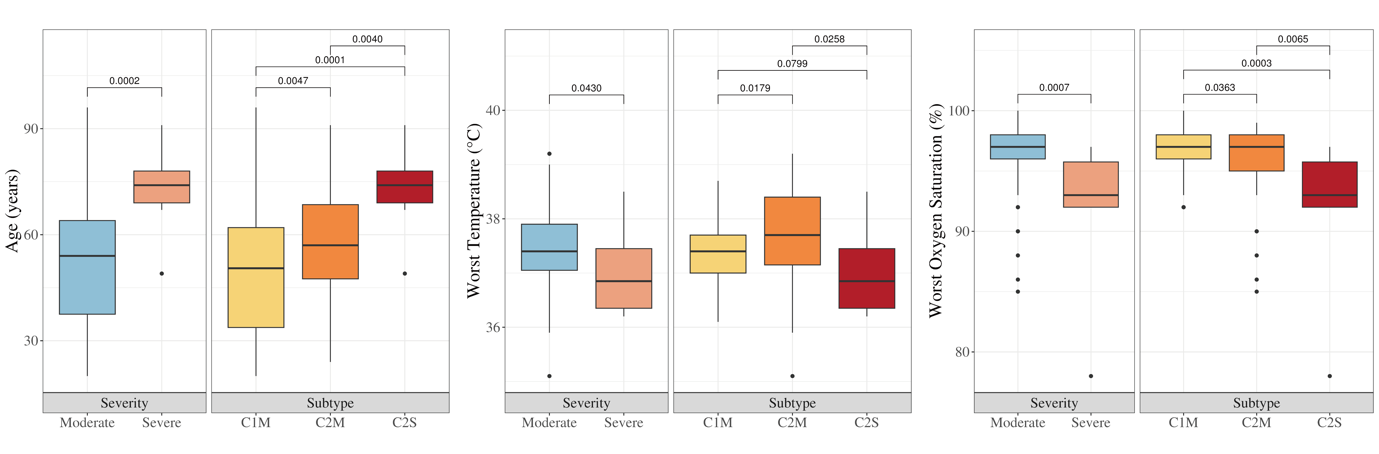


Supplementary Figure 2. Comparison of continuous clinical variables across patient clusters. Boxplots show the distributions of age, worst recorded body temperature, and worst recorded oxygen saturation levels for patients in the Moderate and Severe severity categories, as well as the C1M, C2M, and C2S subtypes.
